# Durability of Viscoelastic Fibre Prestressing in a Polymeric Composite

**DOI:** 10.3390/polym15040811

**Published:** 2023-02-06

**Authors:** Xueqi Lin, Bing Wang, Chenmin Zhao, Walter Nsengiyumva, Shuncong Zhong, Hui Chen, Dianzi Liu

**Affiliations:** 1 Fujian Provincial Key Laboratory of Terahertz Functional Devices and Intelligent Sensing, School of Mechanical Engineering and Automation, Fuzhou University, Fuzhou 350108, China; 2 Institute of Precision Instrument and Intelligent Measurement & Control, Fuzhou University, Fuzhou 350108, China; 3 School of Engineering, University of Hull, Hull HU6 7RX, UK; 4 Engineering Division, Faculty of Science, University of East Anglia, Norwich NR4 7TJ, UK

**Keywords:** durability, polymeric composite, viscoelasticity, prestress, impact

## Abstract

Viscoelastic fibre prestressing (VFP) is a promising technique to counterbalance the potential thermal residual stress within a polymeric composite, offering superior mechanical benefits for structural engineering applications. It has been demonstrated that the time required for a desirable creep strain can be significantly reduced by implementing higher creep stress, while its long-term stability is still unknown. Here, we developed the prestress equivalence principle and investigated the durability of viscoelastic fibre prestressing within a composite in order to further enrich the prestress mechanisms. The effectiveness of the prestress equivalence principle was refined through Charpy impact testing of prestressed samples with various pre-strain levels. The durability was investigated by subjecting samples to both natural aging (up to 0.5 years) and accelerated aging (by using the time-temperature superposition principle). It is found that the prestress equivalence principle offers flexibility for viscoelastically prestressed polymeric matrix composite (VPPMC) technology; the impact benefits offered by VFP are still active after being accelerated aged to an equivalent of 20,000 years at 20 °C, inferring long-term reliability of VFP-generated fibre recovery within a polymeric composite. These findings demonstrated that both materials and energy consumption could be conserved for advanced composites. Therefore, they promote further steps of VPPMC technology toward potential industrial applications, especially for impact protection.

## 1. Introduction

Polymeric matrix composites (PMC) have been widely used in aerospace, automotive, biomedical, as well as sustainable engineering [1,2]. It is known that thermal residual stress levels within a PMC can be significantly affected by the fibre prestressing technique, depending on the fibre and matrix combinations, as well as the laminate stacking sequences [3,4,5]. This can be achieved through (i) elastic fibre prestressing (EFP) [6,7,8] and (ii) viscoelastic fibre prestressing (VFP) [9,10]. For (i), tension is applied to long fibres embedded in an uncured polymeric matrix; the prestress load is released on solidification of the resin to produce an elastically prestressed polymeric matrix composite (EPPMC); whilst for (ii), creep tension is applied to long fibres, then the load is released prior to mould the fibres into a resin; following curing of the matrix, a viscoelastically prestressed polymeric matrix composite (VPPMC) is manufactured [4].

It has been demonstrated that both (i) and (ii) techniques can improve the mechanical properties of polymeric composites without increasing their mass or structural dimensions [10], as well as reducing process-induced deformation [11]. These benefits are induced by improved internal stress levels by either EFP-generated recovery (for EPPMCs) or VFP-generated recovery (for VPPMCs) along the prestressed fibres. Although the fibre prestressing technique is promising, its applications depend on the mechanical properties of the prestressed fibres [12,13]. Commonly, EFP is applicable to brittle (elastic) fibres such as carbon fibres and glass fibres [14,15,16,17], whilst VFP is more suitable for tough fibres such as semi-crystalline thermoplastic fibres [18]. As for structural applications, it is demonstrated that VPPMCs are superior to EPPMCs in terms of product geometry and longevity [19,20]. To produce an EPPMC, the tension load is maintained throughout the curing process, which significantly restricts the product geometry, and equipment designs for simultaneously stretching and moulding can be technically challenging [21,22,23]; whilst for VPPMC, fibre prestressing and moulding are decoupled, providing total flexibility in product geometries [24,25]. As for longevity, VFP-generated fibre recovery has been demonstrated to be a long-term activity [26], while EFP-induced benefits will deteriorate with time due to the localised matrix creep [19,27]. Previous works have shown that the VFP within a PMC can improve the tensile strength by ~15% [20], impact toughness by ~50% [28,29,30,31], and flexural stiffness by ~50% [32]. The VFP-induced benefits have also been applied to produce morphing (bistable) structures [33,34], green composites [35], and have shown great potential for viscoelastically active sutures [36].

The fibre prestressing technique is effective in improving the mechanical properties of PMCs, and the prestress mechanisms have also been introduced. The prestressed fibre recovery generates compressive stresses in the fibre/matrix interface, which in turn interacts with the thermal residual stress [37] to adjust the in-plane stress levels [38]; the fibres are pre-stretched before embedded into a matrix, which is effective in destroying the defective fibres in advance in order to reduce the energy impacts from the stress waves generated by their premature failures on adjacent fibres [39,40]; the fibre prestressing process improves the straightness of the fibre bundles, thus increases the number of effective fibres when loaded in service and improves the load-bearing capacity of a cured composite [15]; impact of a prestressed PMC triggers the interfacial shear stress caused by fibre prestressing, which promotes the interface debonding between fibre and matrix to absorb energy, and thus improves the transverse impact resistance [24,28,29]; the compressive stress generated by the prestressed fibres shifts the neutral axis of the composite under bending, which interacts with the applied stress on the tension surface, hence improves the flexural stiffness of the composite [32].

Although it has been demonstrated that the same VFP benefits could be achieved by exploiting a higher fibre creep stress over a shorter term [30], the long-term reliability of the optimised VFP within a PMC is still unknown. In this research, we develop a prestress equivalence principle and investigate the durability of viscoelastic fibre prestressing within a polymeric composite, aiming to further enrich the underlying prestress mechanisms. Since prestress benefits within a VPPMC depend on the viscoelastic recovery of the prestressed fibres, VFP levels can be represented by viscoelastic creep strain values or ‘pre-strain’ levels, i.e., the effect of elastic strains is subtracted from the total creep strains. The Weibull-based creep and recovery model allows the prediction of viscoelastic creep strain at each optimised creep condition, and this shows opportunities to achieve prestress equivalence between different creep stresses, offering further flexibility for VPPMC technology. The durability of VFP within the PMC is then investigated through both natural aging (up to 6 months) and accelerated aging (using the time-temperature superposition principle) in order to reveal further insight into the fundamental VFP mechanisms.

## 2. Theoretical

### 2.1. Prestress Equavilence Principle

**Figure 1:**
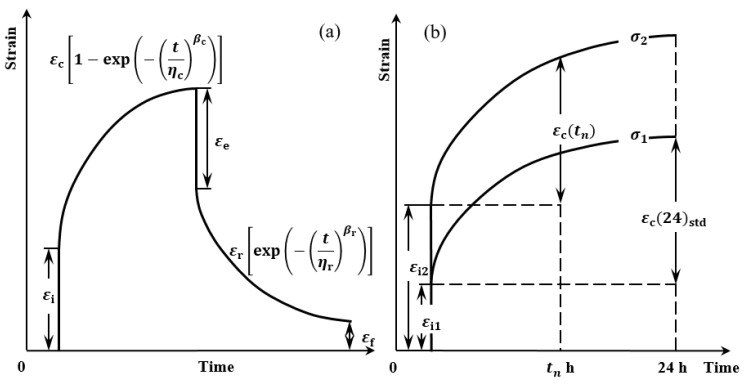
Schematic of the strain-time behaviour of a polymeric fibre under constant creep stress, showing (**a**) creep-recovery strain cycle; and (**b**) the prestress equivalence principle.

When a polymeric fibre is subjected to creep stress (below yield stress), it undergoes both elastic and viscoelastic deformation. The viscoelastic behavior of creep and recovery can be represented by a number of Voigt elements connected in series. Figure 1a shows the strain evolution during the creep and recovery cycle of a polymeric material. The time-dependent components represented by functions are based on the Weibull model [41]. For creep, *ε*_ctot_(*t*) is the total strain at time *t* under an applied constant creep stress:

(1) $$\varepsilon_{\mathrm{ctot}}\left(t\right)=\varepsilon_{i}+\varepsilon_{c}\left[1-\exp\left(-{\left(\frac{t}{\eta_{c}}\right)}^{\beta_{c}}\right)\right]$$

Here, *ε*_i_ is the instantaneous strain from applied stress which is time-independent; *ε*_c_ function is the time-dependent creep strain, where *η*_c_ is the characteristic life, and *β*_c_ is the shape parameter. Following the removal of the creep stress, the elastic deformation is recovered, as represented by *ε*_e_ in Figure 1a, and the remaining recovery strain *ε*_rvis_(*t*) is:

(2) $$\varepsilon_{\mathrm{rvis}}\left(t\right)=\varepsilon_{r}\left[\exp\left(-{\left(\frac{t}{\eta_{r}}\right)}^{\beta_{r}}\right)\right]+\varepsilon_{f}$$

The *ε*_r_ function is the time-dependent recovery strain, with *η*_r_ and *β*_r_ being the Weibull parameters analogous to those in Equation (1). The non-recoverable strain from the viscous flow is represented by *ε*_f_ in Figure 1a.

To get similar prestress benefits, the same pre-strain level can be achieved through shorter terms via higher creep stress, and the equivalence principle is illustrated in Figure 1b. Here, fibre creep tension is applied for 24 h for convenience. Since the creep strain curve is represented by Equation (1), the instantaneous strain *ε*_i1_ and the time-dependent strain *ε*_c_(24)_std_, can be determined experimentally under stress *σ*_1_. The pre-strain level is defined as [*ε*_c_(24)_std_ − *ε*_i1_]. To achieve the same pre-strain level, the subsequent run is performed at a higher stress value, *σ*_2_ (>*σ*_1_). For the strain value at *ε*_c_(*t*_n_) to be equal to *ε*_c_(24)_std_, *t*_n_ will be <24 h. Note, *ε*_c_(*t*_n_) excludes the instantaneous strain *ε*_i2_. Therefore, a value for *t*_n_ which approaches the shortest practical creep time, *t*_min_, can be determined to give similar VFP benefits. Thus, the same pre-strain level can be achieved through a shorter term via a higher creep stress level, and the prestress equivalence principle is mathematically:

(3) $$\sigma^{-1}=a\ln t_{n}+b$$

where *a* and *b* are constants depending on the pre-strain level, which can be determined experimentally.

To validate the prestress equivalence principle, VPPMC samples are produced under the *t*_n_ creep conditions. Thus, batches of VPPMC samples using *t*_n_ are compared with similar batches produced under standard (24 h) creep conditions to evaluate the VFP benefits. Viscoelastic force, σ(t), under *t*_n_ creep conditions is also measured and compared to the standard 24 h runs, in order to provide direct experimental evidence and reveal the fundamental VFP mechanisms, which follows [42]:

(4) $$\sigma \left(t\right)=\sigma_{v}\left[\exp\left(-{\left(\frac{\Delta t}{\eta }\right)}^{\beta }\right)-\exp\left(-{\left(\frac{t}{\eta }\right)}^{\beta }\right)\right]$$

where, the *σ*_v_ function represents VFP-generated time-dependent stress, as determined by the characteristic life *η* and shape *β* parameters.

### 2.2. Durability Prediction through TTSP

The time-temperature superposition principle (TTSP) has been commonly used to generate the master curve for tensile creep [43,44,45], flexural creep [46], dynamic tensile modulus [47], stress relaxation [47,48], or predict the long-term viscoelastic polymeric fibre recovery [26]. The TTSP is based on the free volume theory [49,50] and adopted here to investigate the durability of VFP within a polymeric composite. VPPMC samples under *t*_n_ creep conditions are subjected to accelerated aging and impact tested to determine the long-term effectiveness of the VFP to reveal the fundamental mechanisms. These are achieved through transferring an elevated temperature into a time scale shift in terms of free volume [51], which is mathematically expressed as [52]:

(5) $$\log\frac{\eta \left(T\right)}{\eta \left(T_{0}\right)}=\log\alpha_{T}=-\frac{B}{2.303f_{0}}\left(\frac{T-T_{0}}{f_{0}/a_{T}+T-T_{0}}\right)$$

where *α*_T_ is defined as the temperature shift factor; *T*_0_ is arbitrarily chosen as the reference temperature, and *T* is any other temperature; *a*_T_ is the thermal expansion coefficient of the free volume fraction; *f*_0_ is the free volume fraction at reference temperature *T*_0_. This yields the well-known Williams-Landel-Ferry (WLF) equation with *C*_1_ = *B*/(2.303*f*_0_), *C*_2_ = *f*_0_/*a*_T_.

The TTSP in Equation (5) infers a non-linear relationship between temperature and the shift factor log *α*_T_; however, nylon 6,6 fibre shows approximately linear viscoelasticity within the yield creep strain, and the linear superposition principle holds [53]. This corresponds with the following: (i) Howard and Williams [43] applied the TTSP to the creep of oriented nylon 6,6 fibre under anhydrous conditions when a low range of creep stress (10–51 MPa) was adopted, and results show that the shift factor log *α*_T_ was linear to the temperature; (ii) Murayama et al. [48] investigated the applicability of TTSP to the stress relaxation of nylon 6,6 fibre, and a linear relationship between shift factor log *α*_T_ and the temperature was obtained; (iii) similarly, a linear curve was also obtained by Dunell et al. [47] through the investigation into a superposition of stress relaxation and dynamic tensile modulus of nylon 6,6 monofilaments at temperatures between 10 and −100 °C; (iv) the linear relationship was also found with unoriented nylon 6,6 filaments [54] when subjected to small creep strain values. Therefore, rather than the non-linear relation as represented in Equation (5), a simpler linear TTSP was established for oriented nylon 6,6 fibre, which is based on the published data in terms of creep [43] and stress relaxation [48].

The linear regression gives a gradient of 0.09765 °C^−1^ [51], this is comparable to 0.093 as determined by Williams and Bender [54] through the investigation of unoriented nylon 6,6 filaments. This enables log *α*_T_ to be determined at 70 °C relative to 20 °C, and the resulting value is −4.8825. Hence, the viscoelastic activity would be 76,300 times faster at 70 °C relative to 20 °C, i.e., if samples are subjected to 70 °C for 2298 h, the prestressing effect from viscoelastic recovery mechanisms will be aged to an equivalent of 20,000 years at 20 °C, as well as to be ~25 years at 50 °C ambient temperature.

## 3. Experimental

### 3.1. Sample Preparation and Durability Evaluation

Composite sample preparation followed the procedures from previous studies using nylon 6,6 fibres and polyester resin [31]. To concentrate on the prestress effects, a *V*_f_ of ~2.0% was adopted for all composite samples and subjected to Charpy impact testing [30]. The durability of VFP within the composite was evaluated through (i) naturally aged short-term and middle-term impact performance, as well as (ii) long-term impact resistance through accelerated aging. For (i), five batches of VPPMC samples were produced for each creep condition and were either stored at 336 h (2 weeks for short-term) or 4392 h (6 months for middle-term) at room temperature (19–22 °C) and then impact tested. For (ii), samples were stored at room temperature for at least 2 weeks, and then subjected to accelerated aging prior to the impact tests.

**Figure 2:**
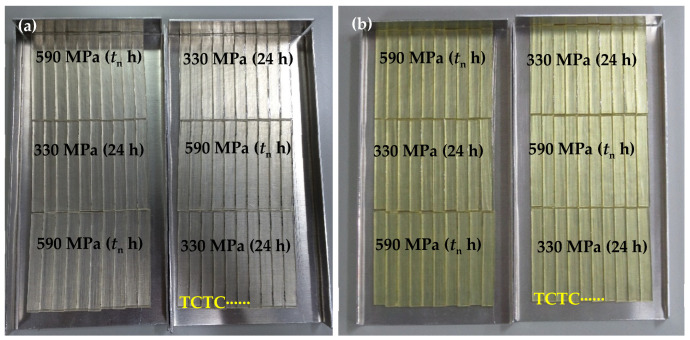
Arrangements of samples within a tray (**a**) before and (**b**) after being subjected to the accelerated aging; “T” stands for test sample, and “C” is the control (non-prestressed) counterpart.

A calibrated fan-assisted oven was used for accelerated aging, with long-term temperature stability of ±0.5 °C. Batches of samples with fibres previously subjected to creep at 590 MPa for *t*_n_ h were aged, together with the standard VPPMC sample batches for comparison. Owning to the limitation in capacity of the fan-assisted oven, three batches of VPPMC samples fabricated with each of the two creep conditions were evaluated (i.e., six batches in total). Heat treatment was maintained at a constant 70 °C for 2298 h (3.2 months), which is equivalent to an exposure of 20,000 years at 20 °C in terms of viscoelastic recovery within the nylon fibres following the TTSP in Section 2.2. Samples were placed as a single layer on the tray, and Figure 2a shows the arrangements of samples before aging, with individual test and control samples in alternating positions. This ensured all the batches were subjected to the same heat conditions. Figure 2b shows the samples after accelerated aging. Samples were removed from the trays and stored in polythene bags at room temperature for a further 336 h prior to impact tests.

### 3.2. Viscoelastic Recovery Force

Previous studies into the force output-time characteristics of viscoelastically prestressed fibres have provided useful evidence for fibre recovery [31,42]. Here, the effectiveness of prestress equivalence principle is studied in terms of recovery force induced by fibre prestressing within the nylon fibre in order to provide further insight into prestress mechanisms. Procedures followed those detailed in [31]. Here, the generated recovery force from up to *t*_n_ creep condition was evaluated and compared to the standard 24 h creep run. Since elastic deformation was fully recovered after load removal, the recovery force induced by the same pre-strain level, i.e., either from the standard 24 h or *t*_n_ h (higher creep stress) run, was expected to be at a similar level.

### 3.3. Statistical Analysis

As stated above, multiple sample batches were tested for various optimised creep conditions for repeatability, which were then compared to the standard 24 h creep run to evaluate the effectiveness of the prestress equivalent principle. Thus, statistical analysis is essential to evaluate the significance level of the data variances. These were performed by following the standard procedures of the two-tailed hypothesis testing at a significant level of 5% [55].

## 4. Results and Discussion

### 4.1. Short-Term Effectiveness

**Figure 3:**
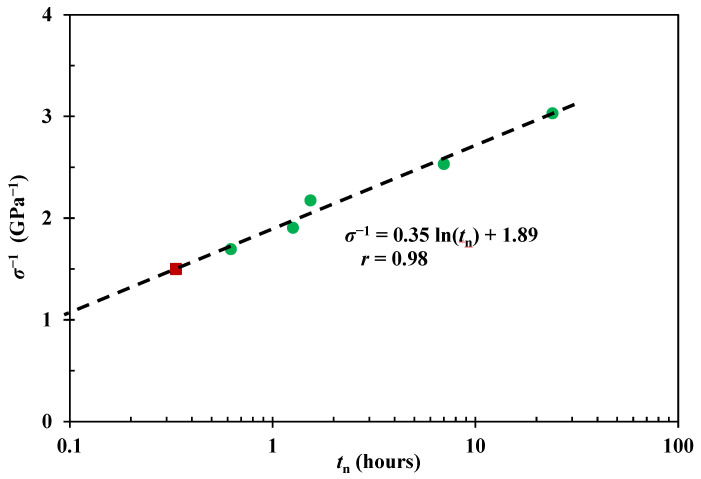
Relationship between applied creep stress *σ* and required *t*_n_ for creep loading to get the same pre-strain level. Round data points are calculated using the prestress equivalence principle in Figure 1b; the square point predicts the creep stress requirement for a stretching period of 20 min; curve is fitted following Equation (3).

Creep and recovery strain-time data of the nylon fibre have been characterised by using the Weibull-based curve-fits from Equation (1), the *ε*_c_(24)_std_ value (330 MPa) was found to be 3.39%. Thus, for *ε*_c_(*t*_n_) to be equal to *ε*_c_(24)_std_, the *t*_n_ values (at ~3.4% pre-strain level) from the 395 MPa, 460 MPa, 525 MPa, and 590 MPa creep data are found to be 420 min, 92 min, 75 min, and 37 min, respectively. Figure 3 shows the applied creep stress vs. *t*_n_ values with fitted logarithmic relationship following Equation (3) at a pre-strain level, *ε*_c_(*t*_n_), of ~3.4%. It offers an opportunity to predict the required creep stress for a designated *t*_n_ value.

The effectiveness of the trend was experimentally evaluated. This was achieved by selecting a point on the extended dashed curve in Figure 3. Batches of Charpy impact samples were made with the obtained creep condition, and benefits from prestress were investigated through impact testing. Equation (3) indicates that stress values of 797 MPa and 715 MPa correspond to 10 min and 15 min stretching respectively; however, initial attempts on creep stress under these conditions rapidly led to fibre fracture due to stress concentration effects. Since it is known that nylon fibre could sustain a 665 MPa creep stress for more than 1 h, this value was adopted here, which requires a corresponding 20 min stretching time to achieve a pre-strain level of ~3.4% in Figure 3. Thus, batches of composite samples were made, and Table 1 shows the impact results (tested at 336 h, i.e., 2 weeks after manufacture). The five batches of samples give an absorbed energy increase of 55.78 ± 3.77%. Two-sided hypothesis testing at a 5% significance level shows that the increase is equivalent to the benefits (54.14 ± 7.52%) from the standard runs (330 MPa for 24 h) [30]. Referring to the creep strain-time data under 665 MPa [56], the strain value *ε*_c_ for *t*_n_ = 20 min was found to be 3.49% using Equation (1), which is comparable to the *ε*_c_(24)_std_ value at 330 MPa, i.e., 3.39%.

These results indicate that ~3.4% viscoelastic creep strain would give an increase of ~56% in absorbed impact energy. In terms of industrial application, this improvement in mechanical properties could be achieved within a few minutes of fibre stretching, as shown in Figure 3, if stress concentration effects can be avoided. It is worth noting that the prestress benefits within a composite may also be related to the stiffness ratio between fibre and matrix, which infers different in-plane stress transfer mechanisms [56].

### 4.2. Recovery Force Equivalence

**Figure 4:**
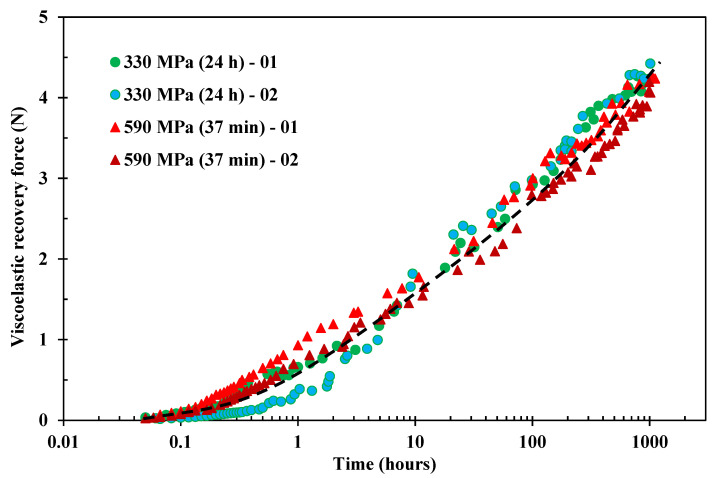
Recovery force measurements from nylon 6,6 fibre after being subjected to the standard creep condition (330 MPa for 24 h) and the 590 MPa 37 min condition.

Further evidence of the prestress equivalence discussed above is to evaluate the recovery force generated by the prestressed fibres. Since the optimised condition in Figure 3 has shown effectively the same mechanical benefits for VPPMCs, the recovery forces with the 590 MPa for 37 min condition were investigated and compared to the standard creep run (330 MPa for 24 h). Two runs were tested for each creep condition (for repeatability), and these were monitored up to 1000 h. Figure 4 shows the resulting data. Although there is variation in data points, the 590 MPa condition shows a slightly higher recovery rate below 10 h, which corresponds with previous findings [31]. This may be due to the quick response of taut-tie molecules (TTMs) upon load removal, which would dominate for several hours. Beyond 10 h however, the 590 MPa condition shows similar recovery force values.

The Weibull-based model represented by Equation (4) was fitted to the experimental data, and corresponding parameter values are presented in Table 2. This enables the prediction of recovery force values at 336 h after creep load removal, which is expected to directly relate to the absolute values of recovery force within a VPPMC at the same age. The resulting data give 3.76 ± 0.06 N and 3.46 ± 0.14 N for 330 MPa and 590 MPa conditions, respectively, and two-tailed hypothesis tests show that these two forces are the same at a significance level of 5%. Thus, the recovery forces generated from the two creep conditions are equivalent within the experimental error. Therefore, it is concluded that recovery force from the reduced time creep (up to 590 MPa creep stress) gives a similar value to the standard creep conditions at an equivalent viscoelastic creep strain level.

Although viscoelastic recovery force and short-term impact tests (336 h) provide evidence of the prestress equivalence, the long-term stability of prestress effects does not necessarily relate to that of a VPPMC due to the possible matrix creep and stress relaxation [19]. Thus, the middle-term and long-term effects of VFP are further investigated and discussed below.

### 4.3. Middle-Term Effectiveness

Furthermore, batches of VPPMC samples with their control counterparts were produced and allowed to age in real-time to 4392 h (0.5 years). Again, batches corresponding to the 590 MPa, 37 min creep condition were evaluated together with standard 330 MPa, 24 h batches for comparison. Table 3 shows the Charpy impact results. Of particular interest is that the increase in impact energy in both cases is comparable, and this is verified through a two-tailed hypothesis testing at a significance level of 5%. Therefore, the viscoelastic recovery mechanism from nylon 6,6 fibre within these VPPMCs is still functional, and there is no deterioration in prestress benefits up to 0.5 years in real-time aging.

### 4.4. Long-Term Effectiveness

When subjected to accelerated aging, apart from the sample colour (yellowish), there is no significant difference visually between the sample batches, see Figure 2b. Table 4 summarises the impact results from tests two weeks after the heat treatment. By subjecting the VPPMC samples to accelerated aging, the increase in impact energy absorption shows that viscoelastically generated prestress remains active for an equivalent of 20,000 years at a constant 20 °C with both creep prestressed samples, i.e., as verified through a two-tailed hypothesis testing (at 5% level), the mean increase value of 52.81 ± 3.09% agrees with the impact result (54.14 ± 7.52%) aged for 336 h. Compared to the optimised creep condition, i.e., 590 MPa for 37 min, the benefits from VFP are also similar. Hypothesis testing shows that the increase of 60.43 ± 9.52% is not different from 52.81 ± 3.09% at a 5% significance level. Hence, there is no deterioration in increased energy absorption.

### 4.5. Durability of Viscoelastic Fibre Prestressing

**Figure 5:**
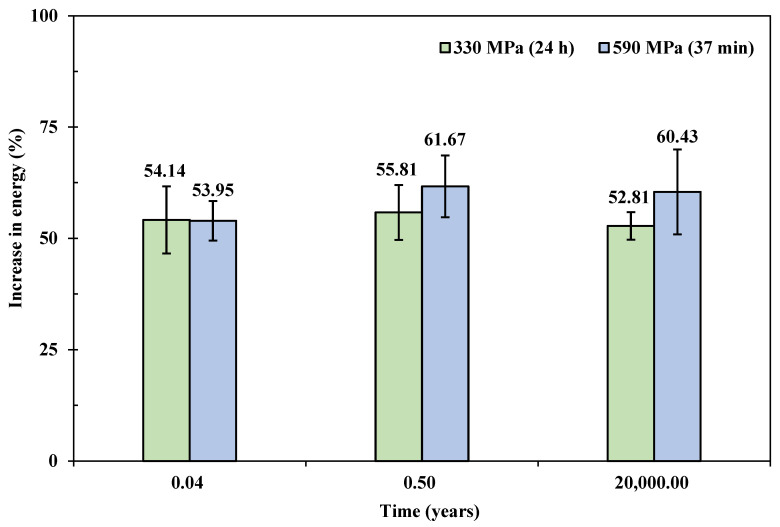
Increase in impact energy against aging time for VPPMC samples produced under different viscoelastic fibre prestressing conditions.

The durability of VFP within the composite is plotted in Figure 5 in terms of Charpy impact resistance. It is clear that the 590 MPa, 37 min creep-conditioned VPPMC samples show a comparable increase in absorbed impact energy with the standard 24 h creep condition (330 MPa) in terms of both natural aging and accelerated aging. This demonstrates that the same pre-strain level achieved through higher creep stress via a shorter term remains active, and there is no deterioration in increased energy absorption in either naturally aged samples up to 0.5 years or accelerated aged to an equivalent of 20,000 years at room temperature. For a VPPMC produced with a viscoelastic creep strain level of ~3.4%, the prestress effects can be expected not to deteriorate for at least ~25 years at a constant 50 °C. This would lead VPPMCs to many practical industrial applications.

Therefore, the effects of nylon fibres prestressed through 590 MPa for 37 min is broadly the same with the 330 MPa, 24 h creep condition within a composite. That is to say, VFP processing time could be reduced from 24 h to tens of minutes by using higher creep stress (below failure stress) with no detriment to impact performance. It is also extrapolated that the logarithmic regression shown in Figure 3 is effective in the long term. Thus, it is concluded that the prestress equivalence principle can be achieved by controlling the viscoelastic creep strain values, i.e., the same pre-strain level would generate equivalent mechanical benefits within a polymeric composite.

### 4.6. Towards Full Potential of Viscoelastic Fibre Prestressing

It has been demonstrated that the benefits from VFP are maximised when fibres are subjected to 460 MPa for 24 h, and the *ε*_c_(24) value (460 MPa) is found to be 4.03% [31]. Thus, further optimisation in load-time conditions could be achieved by applying the prestress equivalence principle in Section 2.1. The prestress benefits can be explored towards the full potential by applying the same principle to a higher viscoelastic creep strain, i.e., ~4.0%. Since it is known that nylon 6,6 can sustain a 665 MPa creep stress for more than 1 h, then for *ε*_c_(*t*_n_) equals *ε*_c_(24) at 460 MPa, the *t*_n_ values for creep at 590 MPa and 665 MPa were found to be 134 min and 48 min, respectively. Data from corresponding Charpy impact results are summarised in Table 5. For reference, batches subjected to 460 MPa for 24 h from ref. [31] are also presented.

**Figure 6:**
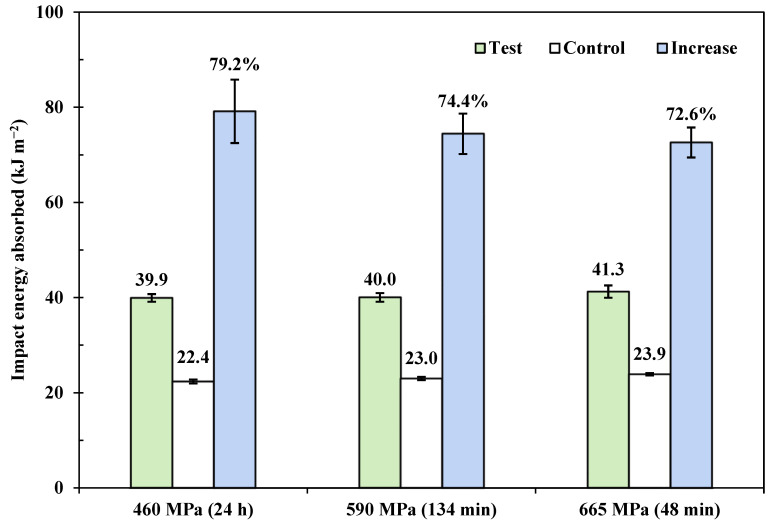
Charpy impact test results, the three creep conditions are equivalent in terms of final viscoelastic creep strain for *ε*_c_(24) at 460 MPa, i.e., ~4.0%; error bars represent the standard error.

The resulting mean data from Table 5 are shown in Figure 6 for comparison. Again, the optimised conditions are in good agreement with the 24 h creep runs in terms of an increase in impact energy absorption. Although there is a slight decrease in increased impact energy over the three creep conditions, from 79% to 73%, little difference was observed in impact energy absorption (in absolute terms). In fact, two-tailed hypothesis testing results show that there is no significant difference in absorbed energy increase, i.e., they are similar. Compared to the creep strain level at ~3.4%, raising the viscoelastic creep strain level to ~4.0% gives a mean increase in energy absorption of ~75%. Thus, an 18% increase in pre-strain level results in a further increase of ~34% in prestress benefits.

A further five batches of VPPMC samples with their control counterparts, produced with the 590 MPa, 134 min creep condition were stored for 4392 h (0.5 years) prior to impact testing to investigate stability in prestress benefits. The results are also shown in Table 5. Again, all batches show effectively the same mechanical benefits from VFP (at a 5% significance level). Therefore, it is concluded that there is no deterioration in impact performance of VPPMC samples at a ~4.0% creep strain level for up to 0.5 years in real-time.

### 4.7. Viscoelastic Fibre Prestressing Mechanisms

**Figure 7:**
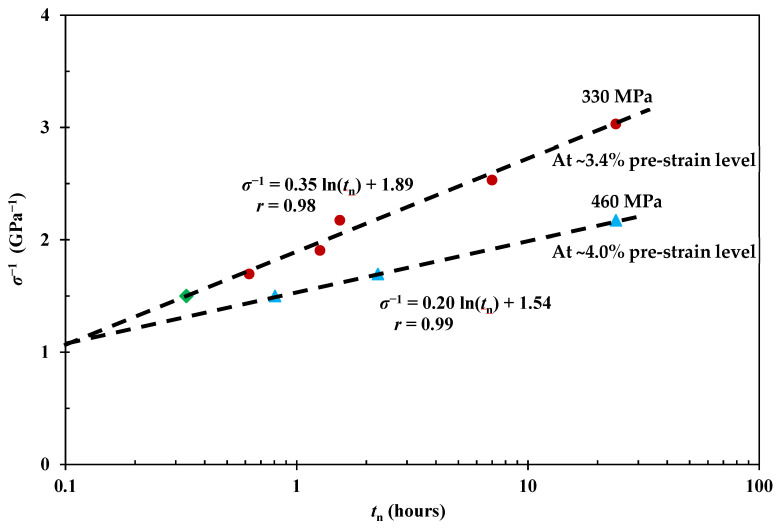
Relationship between applied creep stress *σ* and required *t*_n_ for loading at two viscoelastic creep strain levels; dashed lines are fitted by using Equation (3).

Figure 7 shows the relationship between applied stress *σ* and *t*_n_ at a pre-strain level of ~4.0% following the principle described in Figure 1, together with the curve at ~3.4% pre-strain level from Figure 3, and fitted by Equation (3). It shows that the two curve fits meet at ~0.1 h with a creep stress of ~920 MPa, which infers that the fibre breaks after a very short time loading. Since the breaking strength of nylon fibre is up to 900 MPa, again, Equation (3) indicates reasonable predictions on prestress equivalence.

Here, two viscoelastic creep strain levels were adopted, where a ~3.4% (Section 4.1) pre-strain level represents the standard creep condition (330 MPa for 24 h), and ~4.0% represents the strain when the maximum mechanical benefits were observed for all the prestress conditions under investigation. For ~3.4%, the impact benefits from the optimised VPPMC samples are broadly the same as the standard prestressed samples (330 MPa for 24 h). Additionally, there is no detriment to impact behaviour in samples either naturally aged to 0.5 years or artificially aged to an equivalent (in terms of TTSP) of 20,000 years at a constant 20 °C. This infers the long-term reliability of VFP in a PMC. Confirmation of effectiveness is also demonstrated at a viscoelastic creep strain (pre-strain) of ~4.0%, where prestress equivalence is observed from the impact performance of VPPMC samples naturally aged up to 0.5 years in real-time. The effect of viscoelastic creep strain level has shown a decrease in benefits when fibres are subjected to 590 MPa for 24 h, i.e., ~4.8% in viscoelastic creep strain [31]. These findings further developed the understanding of the viscoelastic prestress mechanisms within the composite.

**Figure 8:**
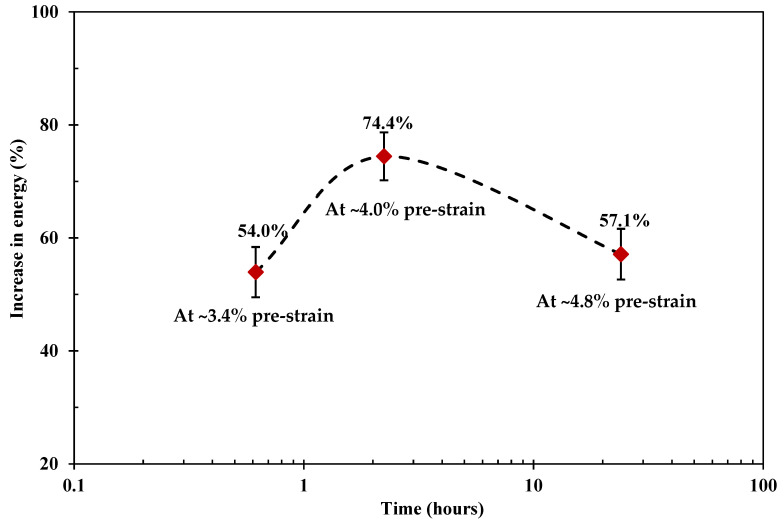
Summary of increase in impact energy from VPPMC samples under a creep stress of 590 MPa. Viscoelastic creep strain levels are achieved through creep exposure, i.e., 37 min for ~3.4%, 134 min for ~4.0% and 24 h for 4.8%.

The VFP mechanisms are closely related to the microstructures of the prestressed fibres. For a viscoelastic solid, the time-dependent deformation can be represented by a series of springs and dashpots. These are treated as consisting of elastic energy storage sites (EESTs) and viscoelastic energy storage sites (VESTs) [56]. For a semi-crystalline polymeric fibre, instantaneous elastic deformation is mainly determined by the crystalline regions, i.e., EESTs, whilst viscoelastic deformation is dominated by the amorphous regions, i.e., VESTs. As implied by the free volume theory and the TTSP, various pre-strain levels can be achieved under the same creep stress for different durations. Figure 8 shows the increase in impact energy under a creep stress of 590 MPa, versus the VFP processing time. When subjected to a constant creep stress, VESTs are progressively triggered to store energy. As the creep stretching progresses over longer periods, an optimum level for prestress benefits from stored energy release was observed. Thus effectively, increasing the creep stress or increasing the creep time has similar effects on VESTs, which controls the mechanical performance of VPPMCs, as demonstrated in Figure 7. This also provides a theoretical foundation for the fact that time-stress superposition principle could be well fitted to the strain-time data of nylon 6,6 fibre [44], i.e., stress effects can be represented by time duration at a constant stress value.

## 5. Conclusions

This research developed the prestress equivalence principle, and investigated the durability of viscoelastic fibre prestressing within a composite, in order to achieve towards the full potential of prestress benefits for VPPMC technology and further enrich the underlying prestress mechanisms. The effectiveness of the prestress equivalence principle was refined through Charpy impact testing of prestressed samples with different pre-strain levels; durability was analysed by subjecting VPPMC samples to both natural aging (up to 0.5 years) and accelerated aging (by applying the time-temperature superposition principle). The VFP mechanisms were then proposed. The main findings include:

(i) The durability of viscoelastic fibre prestressing in a polymeric composite is evaluated through both natural aging (up to 0.5 years) and accelerated aging. It is found that the impact benefits are still active after being accelerated aged to an equivalent of 20,000 years at 20 °C, inferring long-term reliability of VFP-generated fibre recovery within a composite;
(ii) The developed prestress equivalence principle shows a logarithmic relationship between applied creep stress *σ* and *t*_n_, allowing the prediction of the *t*_n_ value required for a given *σ*. This is further verified by exploiting various creep stress conditions to obtain the same pre-strain level. It can also be applied to pursue towards achieving the full mechanical potential of the viscoelastic fibre prestressing, indicating further flexibility for VPPMC technology;
(iii) Longer exposure of nylon 6,6 yarns to a higher strain level could increase the viscoelastic prestress-induced mechanical benefits. Compared to the ~3.4% pre-strain level, an 18% increase in viscoelastic creep strain results in a ~34% increase in prestress benefits, and there is no deterioration in prestress benefits up to 0.5 years in real-time;
(iv) The increase in impact energy is a function of creep time at a constant creep stress (590 MPa). There is an optimum pre-strain level to maximise the prestress benefits. Increasing the creep stress (at constant creep time) or the creep time (at constant creep stress) have similar effects on the microstructures of the prestressed fibres; the prestress mechanisms are subsequently proposed.

These findings demonstrated that both materials and energy consumption could be conserved for manufacturing advanced composites and the viscoelastic fibre prestressing presents long-term reliability within a polymeric composite. Therefore, they promote further steps of VPPMC technology toward potential industrial applications, especially for impact protection.

## Figures and Tables

**Table 1:** Charpy impact results for VPPMC samples produced with a creep condition of 665 MPa for 20 min; tests were performed at 336 h after manufacture; SE is the standard error.

	Natural Age (h)	Mean Impact Energy (kJ m^−2^)		Increase in Energy (%)
Batch		Test ± SE	Control ± SE	
330 MPa (24 h)	336	33.93 ± 3.14	23.78 ± 1.48	42.64
		37.02 ± 1.78	20.71 ± 0.63	78.76
		35.36 ± 1.71	25.03 ± 0.96	41.26
		36.71 ± 2.89	25.60 ± 1.15	43.37
		35.58 ± 1.96	21.61 ± 1.13	64.69
Mean ± SE		35.7 ± 1.0	23.4 ± 0.6	54.1 ± 7.5
665 MPa (20 min)	336	34.74 ± 1.18	22.54 ± 1.16	54.14
		37.32 ± 1.44	24.00 ± 0.59	55.54
		38.42 ± 1.78	22.73 ± 0.45	68.99
		38.74 ± 2.46	25.03 ± 0.59	54.79
		34.00 ± 1.69	23.38 ± 0.55	45.46
Mean ± SE		36.65 ± 0.82	23.53 ± 0.35	55.78 ± 3.77

**Table 2:** Summary of the viscoelastic recovery force parameter values using Equation (4); *r* is the correlation coefficient.

Creep Condition	Recovery Force				
	*σ*_v_ (MPa)	Δ*t* (h)	*η* (h)	*β*	*r*
330 MPa-24 h-01	5.2028	0.0414	9.6148	0.3382	0.9989
330 MPa-24 h-02	5.0470	0.1632	59.778	0.3831	0.9968
590 MPa-37 min-01	11.744	0.0676	4135.6	0.1337	0.9989
590 MPa-37 min-02	14.527	0.0751	116,460	0.1548	0.9988

**Table 3:** Summary of Charpy impact results, tested at 4392 h (0.5 years) after manufacture. Five sample batches (5 test and 5 control samples in each batch) were tested for each viscoelastic fibre prestressing condition; SE is standard error.

	Natural Age (h)	Mean Impact Energy (kJ m^−2^)		Increase in Energy (%)
Batch		Test ± SE	Control ± SE	
330 MPa (24 h)	4392	40.00 ± 2.40	25.77 ± 0.64	55.21
		35.50 ± 3.12	20.32 ± 0.63	74.72
		37.71 ± 1.44	23.43 ± 0.81	60.96
		34.97 ± 3.71	23.13 ± 0.38	51.20
		34.31 ± 3.01	25.05 ± 1.14	36.95
Mean ± SE		36.50 ± 1.22	23.54 ± 0.47	55.81 ± 6.17
590 MPa (37 min)	4392	44.14 ± 1.82	25.17 ± 1.27	75.33
		44.26 ± 3.11	24.46 ± 0.50	80.98
		39.76 ± 2.70	25.51 ± 0.69	55.85
		35.34 ± 1.74	23.96 ± 1.78	47.49
		34.06 ± 1.90	22.90 ± 0.35	48.71
Mean ± SE		39.51 ± 1.24	24.40 ± 0.50	61.67 ± 6.94

**Table 4:** Summary of Charpy impact results. Samples were subjected to accelerated aging to an equivalent of 20,000 years at 20 °C. Each prestressing condition is represented by three batches, with 5 test and 5 control samples in each batch; SE is standard error.

	Exposure to 70 °C	Age Equivalent @ 20 °C	Mean Impact Energy (kJ m^−2^)		Increase in Energy (%)
Batch			Test ± SE	Control ± SE	
330 MPa (24 h)	2298	20,000	34.51 ± 2.07	22.30 ± 1.11	54.73
			33.74 ± 1.63	22.99 ± 0.77	46.76
			34.77 ± 2.32	22.15 ± 0.96	56.94
Mean ± SE			34.34 ± 1.09	22.48 ± 0.52	52.81 ± 3.09
590 MPa (37 min)	2298	20,000	31.78 ± 0.81	22.44 ± 0.84	41.64
			32.65 ± 0.20	18.93 ± 0.47	72.47
			35.91 ± 1.74	21.48 ± 0.33	67.18
Mean ± SE			33.45 ± 0.76	20.95 ± 0.51	60.43 ± 9.52

**Table 5:** Summary of Charpy impact test results, tested at 336 h and 4392 h after manufacture. Five sample batches were tested for each prestressing condition with 5 test and 5 control samples in each batch; SE is standard error.

	Natural Age (h)	Mean Impact Energy (kJ m^−2^)		Increase in Energy (%)
Batch		Test ± SE	Control ± SE	
460 MPa (24 h)	336	39.63 ± 2.22	24.08 ± 0.74	64.60
		37.30 ± 0.54	22.28 ± 0.88	67.39
		38.57 ± 1.35	19.46 ± 0.15	98.21
		39.36 ± 0.95	22.59 ± 0.66	74.23
		44.81 ± 1.86	23.41 ± 0.59	91.36
Mean ± SE		39.93 ± 0.81	22.36 ± 0.42	79.16 ± 6.66
590 MPa (134 min)	336	38.73 ± 1.76	20.79 ± 0.62	86.26
		39.38 ± 1.19	23.18 ± 0.68	69.90
		38.35 ± 1.95	23.70 ± 0.51	61.82
		42.00 ± 3.15	23.25 ± 0.74	80.69
		41.75 ± 3.39	24.06 ± 1.49	73.53
Mean ± SE		40.04 ± 0.92	22.99 ± 0.35	74.44 ± 4.24
665 MPa (48 min)	336	42.07 ± 4.99	23.91 ± 0.49	75.95
		43.29 ± 1.86	23.87 ± 0.66	81.23
		37.57 ± 1.53	22.81 ± 0.60	64.67
		39.29 ± 2.10	23.66 ± 0.72	66.05
		44.07 ± 2.84	25.16 ± 0.09	75.17
Mean ± SE		41.26 ± 1.30	23.89 ± 0.28	72.61 ± 3.15
590 MPa (134 min)	4392	37.65 ± 2.09	26.05 ± 0.74	44.54
		38.57 ± 2.44	25.14 ± 0.63	53.38
		36.16 ± 3.16	22.82 ± 0.46	58.48
		40.19 ± 3.49	22.09 ± 0.66	81.95
		42.26 ± 4.60	23.20 ± 0.28	82.17
Mean ± SE		38.96 ± 1.21	23.86 ± 0.42	64.10 ± 7.66

## Data Availability

All the results presented in the manuscript could be requested to the corresponding author.

## Abbreviations

EEST	Elastic energy storage site
EFP	Elastic fibre prestressing
EPPMC	Elastically prestressed polymeric matrix composite
PMC	Polymeric matrix composite
SE	Standard error
TTM	Taut-tie molecule
TTSP	Time-temperature superposition principle
VEST	Viscoelastic energy storage site
VFP	Viscoelastic fibre prestressing
VPPMC	Viscoelastically prestressed polymeric matrix composite
WLF	Williams-Landel-Ferry
